# Self-rated functioning in family, social, and social-life domains in relation to reasons for living among young adults: a cross-sectional study

**DOI:** 10.3389/fpubh.2026.1935059

**Published:** 2026-09-09

**Authors:** Agnieszka Nieradko-Heluszko, Aleksandra Rył, Marta Giezek, Beata Karakiewicz

**Affiliations:** 1 Subdepartment of Social Medicine and Public Health, Department of Social Medicine, Pomeranian Medical University, Szczecin, Poland; 2 Department of Medical Rehabilitation and Clinical Physiotherapy, Pomeranian Medical University in Szczecin, Szczecin, Poland

**Keywords:** family functioning, life satisfaction, parenthood, reasons for living, self-efficacy, social functioning, suicide prevention, young adults

## Abstract

**Introduction:**

Suicide prevention research has increasingly emphasized protective factors in addition to risk factors. Reasons for living represent a protective construct in suicidology, reflecting beliefs and commitments that may discourage suicidal behavior. However, less is known about how self-rated functioning in family, social, and social-life domains is associated with reasons for living among young adults.

**Methods:**

An anonymous cross-sectional online survey using non-probability convenience sampling was conducted between March and June 2025 among 546 young adults aged 18–35 years in Poland. Reasons for living were assessed using the Polish adaptation of the Reasons for Living Inventory. Life satisfaction and general self-efficacy were measured using the Satisfaction With Life Scale and General Self-Efficacy Scale. Participants rated family, social, and social-life functioning on five-point scales. Hierarchical multiple linear regression examined associations with reasons for living after accounting for selected sociodemographic and family-related factors, life satisfaction, and general self-efficacy.

**Results:**

Reasons for living were positively correlated with life satisfaction, general self-efficacy, and self-rated functioning in all three domains. Selected sociodemographic and family-related variables explained 8.2% of the variance. Adding life satisfaction and general self-efficacy increased explained variance by 16.0%, and adding family, social, and social-life functioning contributed a further 1.4% (*p* = 0.022). The fully adjusted model explained 25.6% of the variance. Life satisfaction showed the strongest positive standardized association, followed by parenthood and general self-efficacy. Family functioning was also positively associated with reasons for living, whereas social and social-life functioning were not independently associated. In a sensitivity analysis excluding Responsibility to Family and Child-Related Concerns items, the association with family functioning was attenuated and no longer statistically significant, whereas parenthood remained positively associated with the non-family-content outcome.

**Conclusion:**

In this convenience sample, reasons for living were cross-sectionally associated with psychological resources and family-related factors. The family-functioning association was sensitive to family-related RFL-48 content, whereas the parenthood association persisted after this content was excluded. Given the non-probability sampling strategy and predominance of women, the findings should not be interpreted as population estimates for Polish young adults. Causal interpretations require longitudinal research, and intervention-related implications require intervention studies.

## Introduction

Suicide remains a major public health concern and one of the leading causes of death among young people worldwide (1). Although research on suicidality has traditionally focused on risk factors, increasing attention has been devoted to protective factors that may reduce vulnerability and strengthen personal and social resources relevant to adaptation and wellbeing (2, 3).

One of the most widely studied protective constructs in suicidology is reasons for living (RFL). The concept refers to beliefs, values, responsibilities, goals, and future-oriented expectations that discourage suicidal behavior despite psychological distress (4). According to the internal suicide debate perspective, suicidal crises often involve a dynamic conflict between reasons for living and reasons for dying, with the relative strength of these competing motivations influencing suicidal outcomes (5, 6). Evidence indicates that stronger reasons for living are associated with lower levels of suicidal ideation, suicide attempts, and suicide risk across both clinical and non-clinical populations (7).

Contemporary models of suicidal behavior emphasize the importance of interpersonal relationships and social connectedness as protective mechanisms. The Interpersonal Theory of Suicide and the Integrated Motivational–Volitional Model suggest that belongingness, social support, and meaningful interpersonal bonds may reduce vulnerability to suicidal thoughts and behaviors (8, 9). Consistent with these assumptions, empirical studies have shown that greater social support and connectedness are associated with lower suicidality and stronger reasons for living (10, 11).

Perceived family functioning represents an important interpersonal context for the development and maintenance of psychological wellbeing. Previous research indicates that positive family relationships, family cohesion, and supportive family environments are associated with better mental health outcomes and lower suicide risk, whereas family dysfunction increases vulnerability to emotional and behavioral difficulties (12, 13). Similarly, perceived social functioning and positive interpersonal engagement have been linked to psychological wellbeing and reduced suicidality (14, 15).

Existing evidence suggests that interpersonal resources may contribute to suicide prevention partly through their association with reasons for living. Individuals who report stronger family connectedness, greater social support, and more satisfying interpersonal relationships tend to endorse stronger life-oriented beliefs and motivations (10, 11, 16). A previous analysis of the same parent web-based survey showed that psychosocial competencies were associated with reasons for living among young adults (17). Although both studies use the same parent sample and the RFL-48 as the outcome, the earlier study focused on four psychosocial competency domains—interpersonal skills, self-awareness, decision-making, and coping with stress—whereas the present study addresses a distinct research question centered on self-rated functioning in family, social, and social-life domains. In addition, the present analysis examines whether these functioning indicators explain incremental variance in reasons for living beyond selected sociodemographic and family-related factors and the individual psychological resources of life satisfaction and general self-efficacy, which was not the analytic focus of the earlier study. However, despite growing evidence on individual and psychosocial correlates of reasons for living, important gaps remain. First, relatively little is known about the association between self-rated functioning in family, social, and social-life domains and reasons for living in non-clinical samples of young adults. Second, it remains unclear whether broad self-rated functioning in these key interpersonal domains provides information about reasons for living beyond sociodemographic and family-related factors and beyond individual psychological resources relevant to suicide prevention, such as life satisfaction and general self-efficacy. Life satisfaction and general self-efficacy were considered relevant individual psychological resources because they capture complementary aspects of positive adaptation. Life satisfaction reflects a global cognitive evaluation of one's life, and lower life satisfaction has been associated with suicidal ideation among university students (18). General self-efficacy reflects perceived capability to cope effectively with difficult situations; in a community-based adult sample, higher general self-efficacy was associated with lower odds of suicidal ideation (19). Both constructs are therefore conceptually relevant to reasons for living: a more positive appraisal of one's life may support life-oriented beliefs and future expectations, whereas perceived coping capability is conceptually aligned with the Survival and Coping Beliefs domain of the RFL-48 (4). Their inclusion therefore allowed the contribution of self-rated interpersonal functioning to be examined beyond broader evaluations of wellbeing and perceived coping capacity. From a public mental health perspective, clarifying this issue may inform future research examining whether broad self-rated functioning in family, social, and social-life domains should be considered alongside individual psychological resources in studies of suicide prevention among young adults. Young adults represent a particularly important group for such research because young adulthood involves developmental transitions related to identity formation, education, employment, intimate relationships, family roles, and increasing autonomy (20).

The aim of this study was to examine whether self-rated functioning in family, social, and social-life domains was associated with reasons for living among young adults after accounting for selected sociodemographic and family-related factors, life satisfaction, and general self-efficacy. Based on previous research, we expected higher self-rated functioning in these domains to be positively associated with reasons for living at the bivariate level. We further expected that these functioning indicators would contribute additional explanatory value beyond selected sociodemographic and family-related factors, life satisfaction, and general self-efficacy. Because family, social, and social-life functioning are conceptually related and were assessed using broad single-item indicators, their unique associations in the fully adjusted model were examined exploratorily.

## Methods

### Study design and participants

This cross-sectional observational study was reported in accordance with the STROBE recommendations for cross-sectional studies (21). The completed STROBE checklist is provided in Supplementary File 1. The present article reports a secondary analysis of the same parent web-based survey previously reported by Nieradko-Heluszko et al. (17). The earlier publication examined psychosocial competencies, whereas the present analysis addresses a distinct research question focused on self-rated functioning in family, social, and social-life domains in relation to reasons for living, with life satisfaction and general self-efficacy included as additional psychological resources. The target population comprised young adults aged 18–35 years. Eligibility for the predefined age range was established during recruitment. No individual-level age variable was available in the analytic dataset for descriptive or multivariable analysis. The final analytic sample consisted of 546 participants who completed the anonymous online questionnaire. Detailed sociodemographic and family-related characteristics are presented in Table 1.

**Table 1:** Selected sociodemographic and family-related characteristics of the analytic sample (*N* = 546).

Characteristic	*n* (%)/*M* ±SD
Gender	
Female	477 (87.36)
Male	69 (12.64)
Place of origin	
Urban area	396 (72.53)
Rural area	150 (27.47)
Raised by	
Both parents	442 (80.95)
Mother only	88 (16.12)
Father only	8 (1.47)
Grandparents	6 (1.10)
Foster care	2 (0.37)
Relationship status	
Married	214 (39.19)
In a non-marital relationship	172 (31.50)
Single	160 (29.30)
Parenthood	
No current children	309 (56.59)
At least one existing child	237 (43.41)
Suicide in the family	
Yes	106 (19.41)
No	386 (70.70)
Do not know	54 (9.89)
Alcohol problem in the family	
Yes	332 (60.81)
No	177 (32.42)
Do not know	37 (6.78)
Continuous variables	
Material situation (1–5)	3.64 ± 0.83
Housing conditions (1–5)	4.21 ± 0.80
Family violence (0–5)	0.73 ± 1.19

Values are presented as *n* (%) or M ± SD. The table summarizes variables presented descriptively and/or included as covariates in the present analysis. Parenthood was categorized as no current children vs. at least one existing child. Higher scores for material situation and housing conditions indicate more favorable conditions, whereas higher scores for family violence indicate a greater frequency of violence.

#### Study size

Because the present study was a secondary analysis of an existing dataset, the available sample size was fixed by the number of complete eligible questionnaires obtained during the recruitment period of the parent survey; therefore, no *a priori* sample size calculation was performed for the present analysis. The analytic sample included 546 participants. A *post hoc* power analysis was conducted for the incremental contribution of the three self-rated functioning indicators in the final hierarchical regression model. Based on *N* = 546, three tested predictors, 15 predictors in the full model, α = 0.05, and the observed incremental effect size of *f* ^2^ = 0.018, the achieved statistical power was approximately 0.76. A sensitivity analysis indicated that the available sample provided 80% power to detect an incremental effect of approximately *f* ^2^ = 0.020. These *post hoc* calculations were interpreted as descriptive and sensitivity information rather than as evidence establishing the adequacy of the available sample size.

### Measures

Reasons for living were assessed using the Polish adaptation of the Reasons for Living Inventory (RFL-48) (22), which was originally developed by Linehan et al. (4). The instrument measures cognitive and emotional beliefs that discourage suicidal behavior despite psychological distress. It consists of 48 items grouped into six domains: Survival and Coping Beliefs, Responsibility to Family, Child-Related Concerns, Moral Objections, Fear of Social Disapproval, and Fear of Suicide. Responses are rated on a six-point Likert scale ranging from 1 (“not at all important”) to 6 (“extremely important”). Higher scores indicate stronger reasons for living. In the present study, Cronbach's α for the total score was 0.96.

Life satisfaction was measured using the Satisfaction With Life Scale (SWLS) (23) in its Polish adaptation (24). The SWLS consists of five items assessing global cognitive evaluations of one's life satisfaction. Responses are provided on a seven-point Likert scale ranging from 1 (“strongly disagree”) to 7 (“strongly agree”). Higher scores indicate greater life satisfaction. In the present study, Cronbach's α for the total score was 0.91.

General self-efficacy was assessed using the General Self-Efficacy Scale (GSES) (25) in its Polish adaptation (26). The scale consists of 10 items measuring individuals' beliefs in their ability to effectively cope with difficult situations and challenges. Responses are rated on a four-point scale. Higher scores indicate greater perceived self-efficacy. In the present study, Cronbach's α for the total score was 0.94.

An author-designed questionnaire was used to collect sociodemographic, family-related, and psychosocial data. It included items assessing gender, place of origin, relationship status, parenthood, family characteristics, material situation, housing conditions, family violence, alcohol problems and suicide in the family, as well as self-rated functioning in selected life domains. Self-rated functioning was assessed using a common author-developed question asking participants to rate their functioning in six life domains: somatic, psychological, social, occupational, family, and social-life functioning. Responses ranged from 1 (“low functioning”) to 5 (“high functioning”). The common item stem, translated from Polish, was “Please indicate, on a scale from 1 to 5, how you rate your functioning in the following area:” followed by the domain labels somatic, psychological, social, occupational, family, and social-life functioning. The present secondary analysis focused on three interpersonal domains: family functioning, social functioning, and social-life functioning. In the original Polish questionnaire, social functioning and social-life functioning were presented as separate items labeled “funkcjonowanie społeczne” and “funkcjonowanie towarzyskie,” respectively. Because the questionnaire did not provide additional behavioral or conceptual definitions specifying how participants should distinguish social functioning from social-life functioning, these domains were treated as broad respondent-defined single-item ratings rather than as validated measures of social support, belongingness, social network structure, or social participation. Each domain was analyzed separately, and no *post hoc* composite functioning score was calculated. Definitions and coding of study variables are provided in Supplementary Table S1.

### Procedure and ethics

Data were collected in Poland between 1 March and 30 June 2025 using an anonymous online questionnaire administered via Google Forms. The survey link was distributed through universities, social media platforms, and thematic online groups targeting young adults. Recruitment was based on non-probability convenience sampling. Participation was voluntary, and no incentives were offered. Because the survey link was distributed through multiple open online channels and no unique visitor tracking was implemented, the number of individuals who viewed the invitation or accessed the questionnaire could not be determined. Consequently, view, participation, and completion rates could not be calculated. No duplicate-prevention procedures under the control of the research team, such as cookie-based controls, IP-based restrictions, log-file analyses, or login/registration-based controls, were used to identify or prevent multiple submissions. Therefore, unique participation could not be technically verified, and duplicate participation cannot be fully excluded. Response timestamps or other technical indicators were not analyzed to identify implausible or duplicate submissions. Where applicable, reporting of the web-based survey procedure was informed by CHERRIES recommendations (27). The completed CHERRIES checklist is provided in Supplementary File 2.

No personally identifying data, such as names, e-mail addresses, or telephone numbers, were collected. The survey did not use item randomization. The survey did not use adaptive questioning or branching logic. Participants could review and modify their responses before final submission. Mandatory response settings were used for all questionnaire items, preventing submission of forms with missing responses in required fields.

Eligibility criteria included being between 18 and 35 years of age. Respondents outside this age range were not included in the analytic sample. Prior to participation, respondents received detailed information regarding the purpose of the study, the voluntary nature of participation, anonymity, confidentiality of the collected data, and the possibility of withdrawing from the study before submitting the questionnaire without any consequences. Submission of the completed questionnaire was considered to constitute informed consent.

The research protocol was approved by the Bioethics Committee of the Pomeranian Medical University in Szczecin, Poland (Approval No. KB-006.028.2025, 21 February 2025). The study was conducted in accordance with the ethical principles of the Declaration of Helsinki and applicable ethical standards for research involving human participants.

#### Potential sources of bias

Several potential sources of bias were considered. The use of an open online survey and non-probability convenience sampling may have introduced selection bias, including self-selection of individuals interested in mental health, interpersonal functioning, or suicide prevention topics. The anonymous self-report format may have reduced social desirability bias; however, subjective reporting bias cannot be excluded. The marked predominance of women in the sample may also limit the external validity of the findings and may have influenced the observed descriptive patterns and associations. These issues were addressed by transparent reporting of the recruitment procedure, eligibility criteria, final analytic sample, and study limitations.

### Statistical analysis

Statistical analyses were performed using Statistica software (TIBCO Software Inc., Palo Alto, CA, USA). A level of *p* < 0.05 in two-tailed tests was adopted as the criterion for statistical significance. Only complete questionnaires were included in the analyses.

Descriptive statistics were calculated for all study variables. Categorical variables were presented as numbers and percentages, whereas continuous variables were presented as means and standard deviations. Internal consistency of the standardized scales was assessed using Cronbach's alpha coefficient. Distributions of continuous variables were assessed using the Shapiro–Wilk test and additionally inspected using *Q*–*Q* plots, skewness, and kurtosis values.

Spearman's rank correlation coefficients were used to assess associations between reasons for living, life satisfaction, general self-efficacy, and self-rated functioning in family, social, and social-life domains. Spearman's correlations were used because the analysis included ordinal self-rated functioning indicators. In the regression models, the three self-rated functioning indicators were entered as numeric 1–5 predictors, and coefficients therefore represent the expected change in the Reasons for Living total score associated with a one-point increase in self-rated functioning.

The main analytical method was hierarchical multiple linear regression analysis, with the total Reasons for Living score as the dependent variable. Predictors were entered in three blocks. The first block included selected sociodemographic and family-related variables: gender, place of origin, relationship status, parenthood, material situation, housing conditions, family violence, alcohol problem in the family, suicide in the family, and being raised by someone other than both parents. The second block included life satisfaction and general self-efficacy. The third block included self-rated functioning in family, social, and social-life domains. This ordering was used to evaluate whether self-rated functioning explained additional variance in reasons for living beyond selected sociodemographic and family-related factors, life satisfaction, and general self-efficacy. For regression analyses, relationship status was recoded into a binary variable: in a relationship, including marital and non-marital relationships, vs. single as the reference category. Parenthood was coded as at least one existing child vs. no current children, with no current children as the reference category. Original free-text responses indicating one or more existing children were coded as parenthood present, whereas responses indicating no current children were coded as no current children. Although the questionnaire allowed multiple responses to the upbringing item, the analytic dataset contained one mutually exclusive upbringing category per participant. For regression analysis, upbringing was dichotomized as raised by both parents vs. any other upbringing arrangement, with raised by both parents as the reference category. For suicide in the family and alcohol problem in the family, “yes” responses were compared with a combined reference category comprising “no” and “do not know” responses.

Model fit was evaluated using *R*^2^ and adjusted *R*^2^. Changes in explained variance between consecutive models were assessed using Δ*R*^2^ and the F-change statistic. Predictor estimates from all three hierarchical models were summarized using unstandardized regression coefficients (*B*), standard errors (SE), 95% confidence intervals, standardized regression coefficients (β), and *p*-values. Changes in *R*^2^ were interpreted as block-level effect-size indicators, whereas standardized regression coefficients were used to compare the relative magnitude of associations within the final model. Approximate linearity, homoscedasticity, and residual normality were evaluated using residual plots and *Q*–*Q* plots. Multicollinearity was assessed using variance inflation factors (VIF), with values below 5 interpreted as indicating no problematic multicollinearity.

To address potential conceptual overlap between parenthood and self-rated family functioning and the family- and child-related content of the RFL-48, additional sensitivity analyses were conducted. First, a *post hoc* non-family-content outcome was calculated by excluding all seven items of the Responsibility to Family subscale and all three items of the Child-Related Concerns subscale, leaving 38 of the original RFL-48 items. This score was used solely as a sensitivity outcome and was not treated as a separately validated version of the RFL instrument. The hierarchical regression analysis was repeated using this outcome. Second, separate sensitivity models were fitted for the Survival and Coping Beliefs and Moral Objections subscales, which do not contain direct family- or child-related content.

To examine whether treating the 1–5 self-rated functioning indicators as numeric predictors materially influenced the findings, additional sensitivity analyses were conducted in which the functioning indicators were entered as categorical predictors, with category 1 as the reference category. Models using categorical and numeric coding were compared to assess evidence of departure from a linear association. HC3 heteroskedasticity-robust standard errors were also examined for the fully adjusted primary model.

No subgroup or interaction analyses were performed.

## Results

### Participants

During the recruitment period, 546 completed questionnaires meeting the eligibility criteria were submitted and included in the final analysis. Because the survey link was distributed through multiple open online channels, the number of individuals who viewed the invitation or accessed the questionnaire could not be determined. Therefore, view, participation, and completion rates could not be calculated. Because mandatory response settings were used for all questionnaire items, the submitted dataset contained no missing item-level data. All participants met the predefined eligibility criterion of being within the 18–35-year age range.

### Descriptive statistics

Descriptive statistics, reliability coefficients, and correlations among the main study variables are presented in Table 2. The mean total score for reasons for living was 196.90 (SD = 48.47). The mean scores for life satisfaction and general self-efficacy were 20.64 (SD = 6.45) and 29.58 (SD = 5.79), respectively. The mean self-ratings of functioning in family, social, and social-life domains were 3.68 (SD = 1.10), 3.51 (SD = 1.02), and 3.44 (SD = 1.09), respectively.

**Table 2:** Descriptive statistics, reliability coefficients, and correlations among reasons for living, life satisfaction, general self-efficacy, and self-rated functioning in family, social, and social-life domains.

Variable	M	SD	α	1	2	3	4	5	6
1. Reasons for living (RFL total)	196.90	48.47	0.963	—	—	—	—	—	—
2. Life satisfaction (SWLS)	20.64	6.45	0.905	0.38	—	—	—	—	—
3. General self-efficacy (GSES)	29.58	5.79	0.936	0.31	0.50	—	—	—	—
4. Family functioning	3.68	1.10	—	0.30	0.49	0.38	—	—	—
5. Social functioning	3.51	1.02	—	0.26	0.44	0.38	0.61	—	—
6. Social-life functioning	3.44	1.09	—	0.19	0.40	0.32	0.60	0.74	—

Correlation coefficients represent Spearman's rho. Cronbach's alpha coefficients were not calculated for single-item indicators of functioning. Cells above the diagonal were omitted because the correlation matrix is symmetrical. All correlations were statistically significant at *p* < 0.001.

### Correlations

Spearman's rank correlations indicated that reasons for living were positively associated with life satisfaction, general self-efficacy, and self-rated functioning in family, social, and social-life domains. The strongest association with the RFL total score was observed for life satisfaction, followed by general self-efficacy and self-rated functioning in the family domain. The three self-rated functioning indicators were also positively interrelated, with the strongest association observed between social functioning and social-life functioning. All correlations were statistically significant at *p* < 0.001.

### Hierarchical regression analysis

The results of the hierarchical regression analysis are presented in Table 3. Complete predictor estimates across all three hierarchical models are provided in Supplementary Table S2, whereas estimates from the fully adjusted model are presented in Table 4. In Model 1, selected sociodemographic and family-related variables explained 8.2% of the variance in reasons for living (*R*^2^ = 0.082, adjusted *R*^2^ = 0.065). In Model 2, the addition of life satisfaction and general self-efficacy increased the explained variance by 16.0% [Δ*R*^2^ = 0.160, ΔF (2, 533) = 56.39, *p* < 0.001], resulting in *R*^2^ = 0.242 (adjusted *R*^2^ = 0.225). In Model 3, the addition of self-rated functioning in family, social, and social-life domains produced a further 1.4% increase in explained variance [Δ*R*^2^ = 0.014, ΔF(3, 530) = 3.23, *p* = 0.022]. The fully adjusted model explained 25.6% of the variance in reasons for living (*R*^2^ = 0.256, adjusted *R*^2^ = 0.235).

**Table 3:** Hierarchical multiple linear regression models predicting Reasons for Living (RFL total).

Model	*R* ^2^	Adjusted *R*^2^	Δ*R*^2^	Δ*F*	df	*p*
Selected sociodemographic and family-related variables	0.082	0.065	0.082	4.774	10, 535	< 0.001
+ SWLS and GSES	0.242	0.225	0.160	56.391	2, 533	< 0.001
+ Self-rated functioning in family, social, and social-life domains	0.256	0.235	0.014	3.227	3, 530	0.022

Δ*R*^2^ represents the increase in explained variance associated with the addition of each block of predictors. Block 1 included selected sociodemographic and family-related variables, including parenthood. Block 2 additionally included life satisfaction (SWLS) and general self-efficacy (GSES). Block 3 additionally included self-rated functioning in family, social, and social-life domains.

**Table 4:** Final hierarchical regression model predicting Reasons for Living (RFL total).

Predictor	B	SE	95% CI for B	β	*p*	VIF
Male (ref. female)	−5.365	5.673	[−16.51, 5.78]	−0.037	0.345	1.079
Rural origin (ref. urban origin)	1.784	4.196	[−6.46, 10.03]	0.016	0.671	1.066
In a relationship, marital or non-marital (ref. single)	−3.521	4.411	[−12.19, 5.14]	−0.033	0.425	1.224
Parenthood, at least one existing child (ref. no current children)	18.106	4.128	[10.00, 26.22]	0.185	< 0.001	1.271
Material situation	−3.789	2.567	[−8.83, 1.25]	−0.065	0.140	1.364
Housing conditions	3.013	2.596	[−2.09, 8.11]	0.050	0.246	1.317
Family violence	−0.822	1.651	[−4.06, 2.42]	−0.020	0.619	1.161
Alcohol problem in the family (yes)	4.275	3.904	[−3.39, 11.94]	0.043	0.274	1.103
Suicide in the family (yes)	−1.998	4.747	[−11.32, 7.33]	−0.016	0.674	1.071
Raised by someone other than both parents	3.883	4.730	[−5.41, 13.17]	0.031	0.412	1.048
Life satisfaction (SWLS)	1.987	0.374	[1.25, 2.72]	0.265	< 0.001	1.766
General self-efficacy (GSES)	1.373	0.379	[0.63, 2.12]	0.164	< 0.001	1.462
Family functioning	5.176	2.347	[0.57, 9.79]	0.117	0.028	2.018
Social functioning	4.361	2.889	[−1.31, 10.04]	0.092	0.132	2.648
Social-life functioning	−4.094	2.601	[−9.20, 1.01]	−0.092	0.116	2.456

Final model: *R*^2^ = 0.256, adjusted *R*^2^ = 0.235. CI = confidence interval for the unstandardized coefficient B. VIF values below 5 indicated no evidence of problematic multicollinearity. For relationship status, married and non-marital relationship categories were combined and compared with single participants as the reference category. Parenthood was coded as at least one existing child vs. no current children, with no current children as the reference category. For alcohol problems and suicide in the family, “yes” was compared with the combined “no/do not know” reference category.

In the fully adjusted model, life satisfaction showed the strongest positive standardized association with reasons for living, followed by parenthood and general self-efficacy. Self-rated family functioning also remained positively associated with the RFL-48 total score, whereas self-rated social functioning and self-rated social-life functioning were not independently associated with the outcome. Among the selected sociodemographic and family-related variables, only parenthood remained statistically significant. Variance inflation factors indicated no evidence of problematic multicollinearity.

### Sensitivity analyses

To examine potential conceptual overlap between the family-related predictors and the family- and child-related content of the RFL-48, the fully adjusted hierarchical model was repeated using a *post hoc* 38-item non-family-content outcome excluding the Responsibility to Family and Child-Related Concerns items. In this sensitivity model, the incremental contribution of the three self-rated functioning indicators was reduced and was no longer statistically significant [Δ*R*^2^ = 0.010, ΔF(3, 530) = 2.41, *p* = 0.066]. Self-rated family functioning was attenuated and no longer independently associated with the outcome [B = 3.14, β = 0.088, *p* = 0.097]. In contrast, parenthood remained positively associated with the non-family-content outcome [B = 11.41, β = 0.145, *p* < 0.001].

Additional analyses using the established non-family RFL-48 subscales showed that the association of family functioning was not consistently reproduced across outcome domains. For Survival and Coping Beliefs, the functioning block explained an additional 1.4% of variance [Δ*R*^2^ = 0.014, ΔF(3, 530) = 3.65, *p* = 0.013], although family functioning itself did not reach conventional statistical significance in the fully adjusted model (B = 2.63, β = 0.097, *p* = 0.061). For Moral Objections, the functioning block did not explain additional variance [Δ*R*^2^ = 0.003, ΔF(3, 530) = 0.55, *p* = 0.646]. Detailed sensitivity estimates are presented in Supplementary Table S3.

Sensitivity analyses addressing the ordinal 1–5 functioning ratings did not indicate a material change in the substantive conclusions when the functioning indicators were treated as categorical rather than numeric predictors. The use of HC3 heteroskedasticity-robust standard errors likewise did not materially alter the inference from the fully adjusted primary model.

## Discussion

### Self-rated family, social, and social-life functioning in relation to reasons for living

The present study examined whether self-rated functioning in family, social, and social-life domains was associated with reasons for living among young adults after accounting for selected sociodemographic and family-related factors, life satisfaction, and general self-efficacy. In the primary fully adjusted model, self-rated family functioning was positively associated with the RFL-48 total score, whereas self-rated social and social-life functioning did not retain statistically significant unique associations. However, the sensitivity analyses showed that the association with family functioning was attenuated and no longer statistically significant after family- and child-related RFL items were excluded from the outcome. Life satisfaction showed the strongest positive association, followed by parenthood and general self-efficacy. Parenthood also remained positively associated with reasons for living after adjustment for the remaining covariates. Taken together, the primary and sensitivity analyses suggest that reasons for living were associated with individual psychological resources and parenthood, whereas the association with self-rated family functioning was more sensitive to the specific content of the RFL outcome. This finding is important because previous suicidological research has more often examined family and social factors in relation to suicidal ideation, suicide attempts, self-harm, or suicide risk than in relation to positive protective constructs such as reasons for living. A substantial body of evidence indicates that poorer family functioning, family dysfunction, and weaker family connectedness are associated with higher suicidality among adolescents and young adults (12, 28–33). Similarly, social connectedness, belongingness, and social support have been associated with lower suicidal ideation and lower suicide risk across different populations (34–41). The present study adds to this literature by focusing on reasons for living rather than on suicidality alone.

### Reasons for living in relation to family and social domains

Reasons for living should not be interpreted simply as the absence of suicidal ideation. They refer to beliefs, values, responsibilities, commitments, and future-oriented expectations that may discourage suicidal behavior despite psychological distress (2, 4, 7). From this perspective, the positive association between self-rated family functioning and the RFL-48 total score is theoretically plausible, particularly because the RFL-48 includes family-related beliefs and responsibilities within its content (4, 22). However, the sensitivity analyses indicate that this association should be interpreted cautiously. When the Responsibility to Family and Child-Related Concerns items were excluded from the outcome, the association with family functioning was attenuated and was no longer statistically significant. Family functioning also did not reach conventional statistical significance in the analyses of Survival and Coping Beliefs and Moral Objections. Taken together, these findings suggest that the association between self-rated family functioning and the overall RFL-48 score may be partly dependent on the family-related content of the instrument rather than representing a consistent association across non-family reasons-for-living domains.

Parenthood showed a different pattern. Although the RFL-48 includes Child-Related Concerns and Responsibility to Family content (4, 22), parenthood remained positively associated with the *post hoc* non-family-content outcome after these items were removed. This reduces, although does not eliminate, concern that the association with parenthood is explained solely by direct content overlap with the RFL-48. Nevertheless, the cross-sectional design does not permit causal or protective interpretations, and the associations observed for individual non-family RFL subscales were not uniform.

The findings are also consistent with studies showing that reasons for living are related to broader psychosocial resources. Social support has been associated with stronger reasons for living among young people (10, 11, 42), while reasons for living have been linked to hope, lower hopelessness, depressive symptomatology, and suicide attempt history (43–47). The present study extends this evidence by examining whether self-rated interpersonal functioning contributed to reasons for living beyond life satisfaction and general self-efficacy; however, the association with family functioning observed for the RFL-48 total score was attenuated in the non-family-content sensitivity analysis.

### Individual wellbeing and self-efficacy in the model

Life satisfaction was the strongest positive correlate of reasons for living in the fully adjusted model, followed by parenthood and general self-efficacy. This is consistent with the theoretical meaning of the RFL construct. Life satisfaction reflects a global cognitive evaluation of one's life, whereas general self-efficacy reflects confidence in one's ability to cope with difficult situations. Both constructs are closely related to the cognitive and motivational components of reasons for living. Previous studies have linked lower life satisfaction with suicidal ideation, suicide attempts, and suicide mortality (48–53).

At the same time, although self-rated family functioning remained positively associated with the RFL-48 total score in the primary model after adjustment for life satisfaction, general self-efficacy, and the remaining covariates, this association was attenuated and no longer statistically significant in the non-family-content sensitivity analysis. This pattern indicates that the family-functioning finding should not be interpreted as a stable independent association across alternative outcome definitions. In contrast, parenthood remained positively associated with the *post hoc* non-family-content outcome. Thus, individual psychological resources and parenthood showed more consistent associations with reasons for living, whereas the association with self-rated family functioning was more sensitive to the specific content of the RFL outcome.

### Implications for public health and preventive practice

The findings do not establish that modifying family, social, or social-life functioning would increase reasons for living. Accordingly, any potential public health relevance of these findings should be regarded as exploratory, and the findings require replication in more representative samples. Causal interpretations require longitudinal research, whereas intervention-related implications require intervention studies using validated multidimensional measures.

### Limitations and directions for further research

Several limitations should be acknowledged. First, the cross-sectional design precludes causal interpretation of the observed associations. It cannot be determined whether self-rated functioning influences reasons for living, whether reasons for living influence subjective evaluations of functioning, or whether both are shaped by unmeasured factors. Age was used only to establish eligibility within the predefined 18–35-year range and was not available as an individual-level analytic variable; therefore, age-related heterogeneity within young adulthood could not be examined.

Second, the study used an online non-probability convenience sample. Response rates and non-participation patterns could not be assessed, and the open anonymous survey design did not allow unique participation to be technically verified. The predominance of women should also be considered, as gender differences in survey participation and suicide-related behaviors may have influenced the observed patterns (54, 55). Consequently, the findings should not be interpreted as population estimates for Polish young adults, and their applicability should be considered primarily in relation to samples with similar recruitment and demographic characteristics.

Third, all variables were assessed using self-report measures, which may have increased common method bias and socially desirable responding. Family, social, and social-life functioning were measured using broad author-developed single-item ratings rather than validated multidimensional instruments. Such single-item ratings provide limited coverage of these complex domains and may be more susceptible to measurement error and subjective interpretation than validated multidimensional measures. In particular, although social functioning and social-life functioning were assessed as separate items using the original Polish labels “funkcjonowanie społeczne” and “funkcjonowanie towarzyskie,” the questionnaire did not provide behavioral or conceptual definitions distinguishing the two domains. Their strong bivariate association (Spearman's ρ = 0.74) further indicates substantial conceptual overlap between the two indicators. Consistent with this, neither social functioning nor social-life functioning retained a statistically significant unique association with reasons for living in the fully adjusted model. Accordingly, the individual regression coefficients for these two indicators should be interpreted cautiously. Future studies should distinguish more explicitly between relationship quality, social participation, perceived support, belongingness, loneliness, and satisfaction with social life, preferably using validated multidimensional measures. In addition, several categorical variables were collapsed for regression analysis, including relationship status and the combination of “no” and “do not know” responses for alcohol problems and suicide in the family, which may have obscured differences between the original categories.

Fourth, because the RFL-48 total score includes family- and child-related content, the associations involving parenthood and self-rated family functioning may partly reflect conceptual overlap with the outcome. The study also did not directly assess suicidal ideation, suicide attempts, self-harm, mental disorders, clinical diagnoses, or psychiatric treatment history. The results should therefore be interpreted in relation to reasons for living as a protective construct rather than as direct evidence of reduced suicide risk.

Finally, the study was conducted in one cultural context, and the final model explained only part of the variance in reasons for living. Future longitudinal research using more diverse samples and validated multidimensional measures is needed to clarify the direction of the observed associations and their relevance to suicide-related outcomes.

## Conclusion

This study indicates that reasons for living among young adults were cross-sectionally associated with life satisfaction, parenthood, general self-efficacy, and self-rated family functioning after adjustment for selected sociodemographic and family-related factors. Life satisfaction showed the strongest positive standardized association, followed by parenthood and general self-efficacy. Although self-rated family functioning was positively associated with the RFL-48 total score in the primary model, this association was attenuated and no longer statistically significant when family- and child-related RFL items were excluded, indicating that the finding is sensitive to conceptual overlap with the outcome. In contrast, parenthood remained positively associated with the *post hoc* non-family-content outcome. These findings underscore the need for caution when interpreting associations between family-related predictors and family-related content within the RFL-48. The cross-sectional design does not establish causal or protective effects. Longitudinal research using more diverse samples and validated multidimensional measures of interpersonal functioning is needed to clarify these associations and their relevance to suicide-related outcomes.

## Data Availability

The data analyzed in this study is subject to the following licenses/restrictions: the de-identified dataset is not publicly available because of ethical and data-protection restrictions. It may be made available by the corresponding author upon reasonable request, subject to applicable ethical and data-protection requirements. Requests to access these datasets should be directed to Agnieszka Nieradko-Heluszko, agnieszka.nieradko.heluszko@pum.edu.pl.
