# Supplementary material for: Self-rated functioning in family, social, and social-life domains in relation to reasons for living among young adults: a cross-sectional study
Source: Front Public Health. 2026 Sep 9;14:1935059. doi: 10.3389/fpubh.2026.1935059 (PMC13597380; doi:10.3389/fpubh.2026.1935059)
Supplement: Supplementary file 1 [file Supplementary_file_1.docx]

**Supplementary File 1. STROBE Statement Checklist for Cross-Sectional Studies**

| **STROBE item** | **Status / completed** | **Location in manuscript** |
| --- | --- | --- |
| 1. Title and abstract | Reported. The title indicates the cross-sectional design and the focus on self-rated functioning in family, social, and social-life domains; the abstract provides an informative summary of what was done and what was found. | Title; Abstract, p. 1 |
| 2. Background/rationale | Reported. The scientific background and rationale for investigating reasons for living in relation to self-rated functioning in family, social, and social-life domains are provided. | Introduction, pp. 2–3 |
| 3. Objectives | Reported. Specific objectives and study expectations are stated, including bivariate associations, incremental explained variance, and exploratory unique associations in the fully adjusted model. | Introduction, final paragraph, p. 3 |
| 4. Study design | Reported. Key elements of the cross-sectional web-based observational design are presented early in the Methods. | Methods: Study design and participants, p. 3 |
| 5. Setting | Reported. The country, data collection period, online survey administration, and recruitment channels are described. | Methods: Procedure and ethics, p. 4 |
| 6. Participants | Reported. Eligibility criteria, target population, recruitment approach, and final analytic sample are described. Participant characteristics are provided in Table 1. | Methods: Study design and participants, p. 3; Procedure and ethics, p. 4; Results: Participants, p. 5; Table 1, p. 3 |
| 7. Variables | Reported. The outcome, main predictors, selected sociodemographic and family-related covariates, and their coding are defined. Additional variable definitions are provided in Supplementary Table S1. | Methods: Measures, pp. 3–4; Statistical analysis, pp. 4–5; Supplementary Table S1, pp. 1–2 |
| 8. Data sources/measurement | Reported. Measurement instruments and author-developed self-rated indicators are described, including response scales and internal consistency for standardized instruments. | Methods: Measures, pp. 3–4 |
| 9. Bias | Reported. Potential selection bias, self-report bias, social desirability bias, and sex/gender imbalance are acknowledged. | Methods: Potential sources of bias, p. 4; Discussion: Limitations and directions for further research, pp. 8–9 |
| 10. Study size | Reported. No formal a priori sample size calculation was performed. The available analytic sample comprised 546 participants. A post hoc power analysis was conducted for the incremental contribution of the three self-rated functioning indicators, together with a sensitivity analysis indicating the incremental effect size detectable with 80% power. | Methods: Study size, p. 3 |
| 11. Quantitative variables | Reported. Handling of continuous and ordinal variables is described. The 1–5 self-rated functioning indicators were treated as numeric predictors in the primary regression models, while Spearman correlations were used for bivariate associations. Additional sensitivity analyses examined categorical coding of the functioning indicators to assess whether treating them as numeric predictors materially influenced the findings. | Methods: Measures, pp. 3–4; Statistical analysis, pp. 4–5; Supplementary Table S1, pp. 1–2 |
| 12. Statistical methods | Reported. Descriptive statistics, internal consistency, Spearman correlations, hierarchical multiple linear regression, model fit, ΔR², F-change statistics, confidence intervals, standardized and unstandardized coefficients, VIF, and residual diagnostics are described. The hierarchical analysis included three sequential blocks. Additional sensitivity analyses addressed potential construct overlap by using a post hoc non-family-content outcome and selected non-family RFL-48 subscales, examined categorical versus numeric coding of the 1–5 functioning indicators, and evaluated HC3 heteroskedasticity-robust standard errors. | Methods: Statistical analysis, pp. 4–5 |
| 13. Participants | Reported. The number of submitted eligible questionnaires and the final analytic sample are reported. Non-participation metrics could not be calculated because the survey link was distributed through open online channels. | Results: Participants, p. 5 |
| 14. Descriptive data | Reported. Selected sociodemographic and family-related characteristics, descriptive statistics, reliability coefficients, and correlations are provided. The dataset contained no missing item-level data. | Results: Descriptive statistics, p. 5; Table 1, p. 3; Table 2, p. 6 |
| 15. Outcome data | Reported. Summary measures for the outcome variable, Reasons for Living total score, are provided. | Results: Descriptive statistics, p. 5; Table 2, p. 6 |
| 16. Main results | Reported. Bivariate correlations, model-fit statistics for all three hierarchical regression models, and complete predictor estimates across Models 1–3 are reported. Unstandardized and standardized coefficients, standard errors, 95% confidence intervals, p-values, and VIF are provided for the fully adjusted model, with complete estimates from all three models presented in Supplementary Table S2. Sensitivity analyses addressing construct overlap, coding of the functioning indicators, and heteroskedasticity-robust inference are reported in the Results and Supplementary Table S3. | Results: Correlations, p. 6; Results: Hierarchical regression analysis, p. 6; Results: Sensitivity analyses, pp. 6–7; Table 2, p. 6; Table 3, p. 6; Table 4, p. 7; Supplementary Table S2, pp. 1–2; Supplementary Table S3, pp. 1–2 |
| 17. Other analyses | Reported. Additional sensitivity analyses were performed to assess potential construct overlap between family-related predictors and the family- and child-related content of the RFL-48, the treatment of the 1–5 functioning indicators as numeric versus categorical predictors, and the robustness of inference using HC3 heteroskedasticity-robust standard errors. No subgroup or interaction analyses were performed. | Methods: Statistical analysis, pp. 4–5; Results: Sensitivity analyses, pp. 6–7 |
| 18. Key results | Reported. The discussion summarizes key findings in relation to the study objectives. | Discussion: Self-rated family, social, and social-life functioning in relation to reasons for living, p. 7 |
| 19. Limitations | Reported. Limitations include the cross-sectional design, online convenience sampling, female predominance, self-report data, single-item functioning indicators, potential conceptual overlap between family- and child-related RFL content and the family-related predictors, lack of direct assessment of suicidality or clinical history, cultural context, and partial explained variance. | Discussion: Limitations and directions for further research, pp. 8–9 |
| 20. Interpretation | Reported. A cautious interpretation is provided in light of study objectives, limitations, previous literature, and the exploratory nature of unique associations in the fully adjusted model. | Discussion, pp. 7–9 |
| 21. Generalisability | Reported. External validity and generalisability limitations are discussed, including sampling strategy, sex/gender imbalance, and cultural context. | Discussion: Limitations and directions for further research, pp. 8–9 |
| 22. Funding | Reported. Funding statement is provided. | Funding, p. 9 |
