# Supplementary material for: Self-rated functioning in family, social, and social-life domains in relation to reasons for living among young adults: a cross-sectional study
Source: Front Public Health. 2026 Sep 9;14:1935059. doi: 10.3389/fpubh.2026.1935059 (PMC13597380; doi:10.3389/fpubh.2026.1935059)
Supplement: Supplementary file 2 [file Supplementary_file_2.docx]

**Supplementary File 2. CHERRIES Checklist for Internet E-Surveys**

| **CHERRIES item** | **Status / completed** | **Location in manuscript** |
| --- | --- | --- |
| **Survey design** | Reported. The study is described as a cross-sectional web-based observational study using an anonymous online questionnaire. | Abstract, p. 1; Methods: Study design and participants, p. 3; Procedure and ethics, p. 4 |
| **Ethics approval** | Reported. Bioethics Committee approval number and approval date are provided. | Methods: Procedure and ethics, p. 4; Ethics statement, p. 9 |
| **Informed consent** | Reported. Participants received study information before participation, and submission of the completed questionnaire constituted informed consent. | Methods: Procedure and ethics, p. 4; Ethics statement, p. 9 |
| **Data protection** | Reported. No personally identifying data, such as names, e-mail addresses, or telephone numbers, were collected; confidentiality and anonymity are described. | Methods: Procedure and ethics, p. 4; Data availability statement, p. 9 |
| **Development and testing** | Partly reported. Standardized instruments and author-developed questionnaire items are described, including the exact wording and response scale of the self-rated functioning question. Information on usability or technical pre-testing of the web questionnaire was not available. | Methods: Measures, pp. 3–4; Supplementary Table S1**, pp. 1**–**2** |
| **Open survey vs. closed survey** | Reported. The survey link was distributed through multiple open online channels; therefore, it was an open web-based survey. | Methods: Procedure and ethics, p. 4 |
| **Contact mode** | Reported. Recruitment was conducted through universities, social media platforms, and thematic online groups targeting young adults. | Methods: Procedure and ethics, p. 4 |
| **Advertising the survey** | Partly reported. The survey link was distributed through universities, social media platforms, and thematic online groups targeting young adults. | Methods: Procedure and ethics, p. 4 |
| **Web survey administration** | Reported. The questionnaire was administered using Google Forms. | Methods: Procedure and ethics, p. 4 |
| **Context** | Reported. The study was conducted in Poland among young adults aged 18–35 years. | Abstract, p. 1; Methods: Study design and participants, p. 3; Procedure and ethics, p. 4 |
| **Voluntary participation** | Reported. Participation was voluntary and participants could withdraw before submitting the questionnaire. | Methods: Procedure and ethics, p. 4 |
| **Incentives** | Reported. No incentives were offered. | Methods: Procedure and ethics, p. 4 |
| **Time/date** | Reported. Data were collected from 1 March to 30 June 2025. | Methods: Procedure and ethics, p. 4 |
| **Randomization of items or questionnaires** | Reported. The survey did not use item randomization. | Methods: Procedure and ethics, p. 4 |
| **Adaptive questioning** | Not used. The questionnaire did not include adaptive questioning or branching logic. | Methods: Procedure and ethics, p. 4 |
| **Number of items and screens/pages** | Partly reported. The questionnaire items are described in the Methods and supplementary materials; the exact number of Google Forms screens was not retained. | Methods: Measures, pp. 3–4; Supplementary Table S1, pp. 1–2 |
| **Completeness check** | Reported. Mandatory response settings were used for all questionnaire items, preventing submission with missing responses in required fields. | Methods: Procedure and ethics, p. 4; Results: Participants, p. 5 |
| **Review step** | Reported. Participants could review and modify their responses before submitting the questionnaire. | Methods: Procedure and ethics, p. 4 |
| **Unique site visitor metrics** | Reported as not available. Because the survey link was openly distributed and no unique visitor tracking was implemented, the number of individuals who viewed the invitation or accessed the questionnaire could not be determined. | Methods: Procedure and ethics, p. 4; Results: Participants, p. 5 |
| **View rate** | Reported as not calculable because unique visitor metrics were unavailable. | Methods: Procedure and ethics, p. 4; Results: Participants, p. 5 |
| **Participation rate** | Reported as not calculable because the number of unique visitors who accessed the questionnaire was unavailable. | Methods: Procedure and ethics, p. 4; Results: Participants, p. 5 |
| **Completion rate** | Reported as not calculable because access and participation denominators were unavailable; the analytic sample included complete eligible submissions. | Methods: Procedure and ethics, p. 4; Results: Participants, p. 5 |
| **Cookies** | No duplicate-prevention procedures under the control of the research team, such as cookie-based controls, IP-based restrictions, log-file analyses, or login/registration-based controls, were used to identify or prevent multiple submissions. | Methods: Procedure and ethics, p. 4 |
| **IP check** | Not performed. IP-based restrictions or checks were not used by the research team. | Methods: Procedure and ethics, p. 4 |
| **Log file analysis** | Not performed. Log files were not analyzed by the research team to identify duplicate entries. | Methods: Procedure and ethics, p. 4 |
| **Registration/log-in** | Not used for duplicate-prevention purposes. No login/registration-based controls were used by the research team to identify or prevent multiple submissions. | Methods: Procedure and ethics, p. 4 |
| **Handling incomplete questionnaires** | Reported. Only complete questionnaires were included; mandatory fields prevented item-level missing data in submitted forms. | Methods: Statistical analysis, p. 5; Results: Participants, p. 5 |
| **Atypical timestamp or duplicate screening** | Not performed. Response timestamps or other technical indicators were not analyzed to identify implausible or duplicate submissions. | Methods: Procedure and ethics, p. 4 |
| **Statistical correction** | Not used. No statistical weighting or correction for sample representativeness was applied. | Not applicable |
