# Supplementary material for: Self-rated functioning in family, social, and social-life domains in relation to reasons for living among young adults: a cross-sectional study
Source: Front Public Health. 2026 Sep 9;14:1935059. doi: 10.3389/fpubh.2026.1935059 (PMC13597380; doi:10.3389/fpubh.2026.1935059)
Supplement: Supplementary file 3 [file Table_1.docx]

**Supplementary Table S1. Definition and coding of study variables**

| **Variable** | **Role in analysis** | **Coding / measurement** |
| --- | --- | --- |
| **Reasons for Living total score** | Dependent variable | Continuous total RFL-48 score; higher scores indicate stronger reasons for living |
| **Gender** | Covariate | Male vs female reference category |
| **Place of origin** | Covariate | Rural origin vs urban origin reference category |
| **Relationship status** | Covariate | In a relationship, including marital and non-marital relationships, vs single reference category |
| **Parenthood** | Covariate | Original free-text responses were coded as at least one existing child versus no current children, with no current children as the reference category. Responses indicating one or more existing children were classified as parenthood present. |
| **Material situation** | Covariate | 1–5 scale; higher scores indicate better material situation |
| **Housing conditions** | Covariate | 1–5 scale; higher scores indicate better housing conditions |
| **Family violence** | Covariate | 0–5 scale; higher scores indicate greater frequency of violence |
| **Alcohol problem in the family** | Covariate | Dummy-coded: yes = 1; no/do not know = 0 reference category |
| **Suicide in the family** | Covariate | Dummy-coded: yes = 1; no/do not know = 0 reference category |
| **Raised by someone other than both parents** | Covariate | Yes vs raised by both parents reference category |
| **Life satisfaction** | Psychological resource / covariate | SWLS total score; higher scores indicate greater life satisfaction |
| **General self-efficacy** | Psychological resource / covariate | GSES total score; higher scores indicate greater general self-efficacy |
| **Family functioning** | Main predictor | 1–5 single-item rating (1 = “low functioning”, 5 = “high functioning”); higher scores indicate better self-rated family functioning; entered as a numeric 1–5 predictor in the primary regression analysis; categorical coding was examined in sensitivity analyses |
| **Social functioning** | Main predictor | 1–5 single-item rating (1 = “low functioning”, 5 = “high functioning”); higher scores indicate better self-rated social functioning; entered as a numeric 1–5 predictor in the primary regression analysis; categorical coding was examined in sensitivity analyses |
| **Social-life functioning** | Main predictor | 1–5 single-item rating (1 = “low functioning”, 5 = “high functioning”); higher scores indicate better self-rated social-life functioning; entered as a numeric 1–5 predictor in the primary regression analysis; categorical coding was examined in sensitivity analyses |

**Note.** Self-rated functioning was assessed using a common author-developed question asking participants to rate their functioning in six life domains: somatic, psychological, social, occupational, family, and social-life functioning. Responses ranged from 1 (“low functioning”) to 5 (“high functioning”). The present secondary analysis included family, social, and social-life functioning as separate single-item predictors; no composite functioning score was calculated. The original Polish labels for social functioning and social-life functioning were “funkcjonowanie społeczne” and “funkcjonowanie towarzyskie,” respectively; no additional behavioral or conceptual definitions were provided to distinguish these two domains.
