# Supplementary material for: Self-rated functioning in family, social, and social-life domains in relation to reasons for living among young adults: a cross-sectional study
Source: Front Public Health. 2026 Sep 9;14:1935059. doi: 10.3389/fpubh.2026.1935059 (PMC13597380; doi:10.3389/fpubh.2026.1935059)
Supplement: Supplementary file 4 [file Table_2.docx]

**Supplementary Table S2. Predictor estimates across the three hierarchical regression models predicting Reasons for Living (RFL-48 total score)**

**Model 1. Selected sociodemographic and family-related variables**

| **Predictor** | **B** | **SE** | **95% CI for B** | **β** | **p** |
| --- | --- | --- | --- | --- | --- |
| Male (ref. female) | −0.485 | 6.207 | [−12.68,11.71] | −0.003 | 0.938 |
| Rural origin (ref. urban origin) | 3.395 | 4.618 | [−5.68, 12.47] | 0.031 | 0.463 |
| In a relationship, marital or non-marital (ref. single) | 1.840 | 4.844 | [−7.68, 11.36] | 0.017 | 0.704 |
| Parenthood, at least one existing child (ref. no current children) | 19.537 | 4.508 | [10.68, 28.39] | 0.200 | < 0.001 |
| Material situation | 2.295 | 2.742 | [−3.09, 7.68] | 0.039 | 0.403 |
| Housing conditions | 7.449 | 2.831 | [1.89, 13.01] | 0.123 | 0.009 |
| Family violence | −3.562 | 1.767 | [−7.03, −0.09] | −0.087 | 0.044 |
| Alcohol problem in the family (yes) | 7.636 | 4.293 | [−0.80, 16.07] | 0.077 | 0.076 |
| Suicide in the family (yes) | −4.554 | 5.234 | [−14.84, 5.73] | −0.037 | 0.385 |
| Raised by someone other than both parents | 4.209 | 5.225 | [−6.05, 14.47] | 0.034 | 0.421 |

**Model fit:** R² = 0.082; adjusted R² = 0.065; F(10, 535) = 4.774; p < 0.001.

**Model 2. Selected sociodemographic and family-related variables + SWLS and GSES**

| **Predictor** | **B** | **SE** | **95% CI for B** | **β** | **p** |
| --- | --- | --- | --- | --- | --- |
| Male (ref. female) | −6.409 | 5.678 | [−17.56, 4.74] | −0.044 | 0.259 |
| Rural origin (ref. urban origin) | 1.597 | 4.209 | [−6.67, 9.86] | 0.015 | 0.704 |
| In a relationship, marital or non-marital (ref. single) | −3.113 | 4.434 | [−11.82, 5.60] | −0.029 | 0.483 |
| Parenthood, at least one existing child (ref. no current children) | 18.994 | 4.108 | [10.92, 27.06] | 0.194 | < 0.001 |
| Material situation | −3.468 | 2.572 | [−8.52, 1.58] | −0.059 | 0.178 |
| Housing conditions | 3.549 | 2.604 | [−1.57, 8.67] | 0.059 | 0.174 |
| Family violence | −1.278 | 1.624 | [−4.47, 1.91] | −0.031 | 0.432 |
| Alcohol problem in the family (yes) | 3.825 | 3.924 | [−3.88, 11.53] | 0.039 | 0.330 |
| Suicide in the family (yes) | −1.844 | 4.773 | [−11.22, 7.53] | −0.015 | 0.699 |
| Raised by someone other than both parents | 3.750 | 4.757 | [−5.59, 13.09] | 0.030 | 0.431 |
| Life satisfaction (SWLS) | 2.308 | 0.352 | [1.62, 3.00] | 0.307 | < 0.001 |
| General self-efficacy (GSES) | 1.593 | 0.372 | [0.86, 2.32] | 0.190 | < 0.001 |

**Model fit:** R² = 0.242; adjusted R² = 0.225; F(12, 533) = 14.200; p < 0.001.

**Model 3. Selected sociodemographic and family-related variables + SWLS and GSES + self-rated functioning in family, social, and social-life domains**

| **Predictor** | **B** | **SE** | **95% CI for B** | **β** | **p** |
| --- | --- | --- | --- | --- | --- |
| Male (ref. female) | −5.365 | 5.673 | [−16.51, 5.78] | −0.037 | 0.345 |
| Rural origin (ref. urban origin) | 1.784 | 4.196 | [−6.46, 10.03] | 0.016 | 0.671 |
| In a relationship, marital or non-marital (ref. single) | −3.521 | 4.411 | [−12.19, 5.14] | −0.033 | 0.425 |
| Parenthood, at least one existing child (ref. no current children) | 18.106 | 4.128 | [10.00, 26.22] | 0.185 | < 0.001 |
| Material situation | −3.789 | 2.567 | [−8.83, 1.25] | −0.065 | 0.140 |
| Housing conditions | 3.013 | 2.596 | [−2.09, 8.11] | 0.050 | 0.246 |
| Family violence | −0.822 | 1.651 | [−4.06, 2.42] | −0.020 | 0.619 |
| Alcohol problem in the family (yes) | 4.275 | 3.904 | [−3.39, 11.94] | 0.043 | 0.274 |
| Suicide in the family (yes) | −1.998 | 4.747 | [−11.32, 7.33] | −0.016 | 0.674 |
| Raised by someone other than both parents | 3.883 | 4.730 | [−5.41, 13.17] | 0.031 | 0.412 |
| Life satisfaction (SWLS) | 1.987 | 0.374 | [1.25, 2.72] | 0.265 | < 0.001 |
| General self-efficacy (GSES) | 1.373 | 0.379 | [0.63, 2.12] | 0.164 | < 0.001 |
| Family functioning | 5.176 | 2.347 | [0.57, 9.79] | 0.117 | 0.028 |
| Social functioning | 4.361 | 2.889 | [−1.31, 10.04] | 0.092 | 0.132 |
| Social-life functioning | −4.094 | 2.601 | [−9.20, 1.01] | −0.092 | 0.116 |

**Model fit:** R² = 0.256; adjusted R² = 0.235; F(15, 530) = 12.148; p < 0.001.

**Note.** B = unstandardized regression coefficient; SE = standard error; CI = confidence interval; β = standardized regression coefficient. Model 1 included selected sociodemographic and family-related variables. Model 2 additionally included life satisfaction (SWLS) and general self-efficacy (GSES). Model 3 additionally included self-rated functioning in family, social, and social-life domains. Relationship status was coded as in a marital or non-marital relationship versus single as the reference category. Parenthood was coded as at least one existing child versus no current children, with no current children as the reference category. For alcohol problem and suicide in the family, “yes” was compared with the combined “no/do not know” reference category.
