# Supplementary material for: Self-rated functioning in family, social, and social-life domains in relation to reasons for living among young adults: a cross-sectional study
Source: Front Public Health. 2026 Sep 9;14:1935059. doi: 10.3389/fpubh.2026.1935059 (PMC13597380; doi:10.3389/fpubh.2026.1935059)
Supplement: Supplementary file 5 [file Table_3.docx]

**Supplementary Table S3. Sensitivity analyses addressing construct overlap, coding of self-rated functioning indicators, and heteroskedasticity-robust inference**

**Panel A. Sensitivity analyses addressing potential construct overlap with family- and child-related RFL-48 content**

| **Sensitivity outcome** | **ΔR² for functioning block** | **ΔF(3, 530)** | **p** | **Family functioning B** | **β** | **p** | **Parenthood B** | **β** | **p** |
| --- | --- | --- | --- | --- | --- | --- | --- | --- | --- |
| Post hoc 38-item non-family-content outcome | 0.010 | 2.409 | 0.066 | 3.138 | 0.088 | 0.097 | 11.407 | 0.145 | < 0.001 |
| Survival and Coping Beliefs | 0.014 | 3.650 | 0.013 | 2.629 | 0.097 | 0.061 | 4.029 | 0.067 | 0.102 |
| Moral Objections | 0.003 | 0.554 | 0.646 | 0.430 | 0.066 | 0.254 | 4.089 | 0.282 | < 0.001 |

**Note.** All sensitivity models used the same hierarchical adjustment strategy as the primary analysis. The post hoc 38-item non-family-content outcome was calculated by excluding all seven Responsibility to Family items and all three Child-Related Concerns items from the RFL-48 and was used solely as a sensitivity outcome, not as a separately validated version of the instrument. Survival and Coping Beliefs and Moral Objections are established RFL-48 subscales without direct family- or child-related item content. ΔR² refers to the incremental variance explained by the block comprising family, social, and social-life functioning.

**Panel B. Sensitivity analysis of numeric versus categorical coding of self-rated functioning indicators**

| **Coding sensitivity model** | **R²** | **Adjusted R²** | **ΔR² vs primary numeric model** | **F test for departure from numeric coding** | **p** |
| --- | --- | --- | --- | --- | --- |
| Family functioning categorical; social and social-life functioning numeric | 0.2565 | 0.2311 | 0.0006 | F(3, 527) = 0.152 | 0.928 |
| Social functioning categorical; family and social-life functioning numeric | 0.2588 | 0.2335 | 0.0029 | F(3, 527) = 0.694 | 0.556 |
| Social-life functioning categorical; family and social functioning numeric | 0.2594 | 0.2342 | 0.0036 | F(3, 527) = 0.854 | 0.465 |
| All three functioning indicators categorical | 0.2682 | 0.2345 | 0.0123 | F(9, 521) = 0.975 | 0.460 |

**Note.** The primary fully adjusted model treated family, social, and social-life functioning as numeric 1–5 predictors (R² = 0.2558; adjusted R² = 0.2348). In the sensitivity models, functioning indicators were represented by categorical dummy variables with category 1 as the reference category. Nested F-tests assessed whether categorical coding provided a significantly better fit than the corresponding numeric coding. For the single-domain comparisons, categorical coding added three degrees of freedom; simultaneous categorical coding of all three functioning indicators added nine degrees of freedom. None of the comparisons provided evidence of a statistically significant departure from the numeric 1–5 specification.

**Panel C. Heteroskedasticity-robust inference for the fully adjusted primary model**

| **Predictor** | **B** | **HC3 robust SE** | **95% CI for B** | **p** |
| --- | --- | --- | --- | --- |
| Male (ref. female) | −5.365 | 5.804 | [−16.77, 6.04] | 0.356 |
| Rural origin (ref. urban origin) | 1.784 | 4.165 | [−6.40, 9.97] | 0.669 |
| In a relationship, marital or non-marital (ref. single) | −3.521 | 4.330 | [−12.03, 4.98] | 0.416 |
| Parenthood, at least one existing child (ref. no current children) | 18.106 | 4.362 | [9.54, 26.68] | < 0.001 |
| Material situation | −3.789 | 2.677 | [−9.05, 1.47] | 0.157 |
| Housing conditions | 3.013 | 2.668 | [−2.23, 8.25] | 0.259 |
| Family violence | −0.822 | 1.547 | [−3.86, 2.22] | 0.596 |
| Alcohol problem in the family (yes) | 4.275 | 4.081 | [−3.74, 12.29] | 0.295 |
| Suicide in the family (yes) | −1.998 | 4.776 | [−11.38, 7.38] | 0.676 |
| Raised by someone other than both parents | 3.883 | 4.740 | [−5.43, 13.19] | 0.413 |
| Life satisfaction (SWLS) | 1.987 | 0.376 | [1.25, 2.73] | < 0.001 |
| General self-efficacy (GSES) | 1.373 | 0.403 | [0.58, 2.17] | < 0.001 |
| Family functioning | 5.176 | 2.310 | [0.64, 9.71] | 0.025 |
| Social functioning | 4.361 | 2.824 | [−1.19, 9.91] | 0.123 |
| Social-life functioning | −4.094 | 2.689 | [−9.38, 1.19] | 0.128 |

**Note.** B = unstandardized regression coefficient; SE = standard error; CI = confidence interval. HC3 heteroskedasticity-consistent standard errors were calculated for the fully adjusted primary model. The regression coefficients are identical to those from the conventional model; robust standard errors, confidence intervals, and p-values are reported here. The substantive pattern of statistical inference was unchanged.
